# P-2128. Intestinal Colonization Screening for Extended-Spectrum Beta-Lactamase-Producing Enterobacterales in Hematopoietic Stem Cell Transplant Recipients: When More Is Not Better

**DOI:** 10.1093/ofid/ofaf695.2292

**Published:** 2026-01-11

**Authors:** Maximiliano Gabriel Castro, Fabián Herrera, Elena Temporiti, Diego Torres, Marcia Querci, Agustina Fiori, Nicolás Lasserre, Julia Ramponi, Gustavo A Castro Torres, Pablo Bonvehí

**Affiliations:** CEMIC: Centro de Educacion Medica e Investigaciones Clinicas Norberto Quirno, Ciudad autónoma de Buenos Aires, Ciudad Autonoma de Buenos Aires, Argentina; Centro de Educación Médica e Investigaciones Clínicas, CEMIC, BUENOS AIRES, Ciudad Autonoma de Buenos Aires, Argentina; Hospital Universitario CEMIC, Buenos Aires, Ciudad Autonoma de Buenos Aires, Argentina; Centro de Educación Médica e Investigaciones Clínicas, CEMIC, BUENOS AIRES, Ciudad Autonoma de Buenos Aires, Argentina; Hospital Universitario CEMIC, Buenos Aires, Ciudad Autonoma de Buenos Aires, Argentina; CEMIC: Centro de Educacion Medica e Investigaciones Clinicas Norberto Quirno, Ciudad autónoma de Buenos Aires, Ciudad Autonoma de Buenos Aires, Argentina; CEMIC: Centro de Educacion Medica e Investigaciones Clinicas Norberto Quirno, Ciudad autónoma de Buenos Aires, Ciudad Autonoma de Buenos Aires, Argentina; CEMIC: Centro de Educacion Medica e Investigaciones Clinicas Norberto Quirno, Ciudad autónoma de Buenos Aires, Ciudad Autonoma de Buenos Aires, Argentina; CEMIC: Centro de Educacion Medica e Investigaciones Clinicas Norberto Quirno, Ciudad autónoma de Buenos Aires, Ciudad Autonoma de Buenos Aires, Argentina; CEMIC: Centro de Educacion Medica e Investigaciones Clinicas Norberto Quirno, Ciudad autónoma de Buenos Aires, Ciudad Autonoma de Buenos Aires, Argentina

## Abstract

**Background:**

Several guidelines recommend rectal carriage screening (RCS) for extended-spectrum beta-lactamase-producing *Enterobacterales* (ESBL-E) to guide empirical antibiotic treatment (EAT) in neutropenic patients. However, this strategy may lead to an unnecessary use of carbapenems (CB). We aimed to evaluate the clinical impact of RCS for ESBL-E on appropriate EAT, CB use, and in-hospital mortality during the first episode of neutropenia in the pre-engraftment period of Hematopoietic Stem Cell Transplantation (HSCT).

**Methods:**

Prospective cohort study of adult hospitalized patients who received HSCT between January 2017 and December 2024, with a 30-day follow-up. Systematic weekly RCS for ESBL-E was performed from the pre-transplant evaluation until hospital discharge. This practice was discontinued in 2023. We compared those who underwent RCS and those who did not (N-RCS). Chi^2^ and Mann–Whitney U tests were performed when appropriate.

**Results:**

318 patients with a median age of 52 years (IQR 30.8–61) were included. Allogeneic HSCT was performed in 33% of cases, of which 34.3% were from unrelated donors and 36.2% were haploidentical. The most common underlying diseases were multiple myeloma (42.5%) and lymphoma (30.2%). *Enterobacterales* bacteremia was diagnosed in 15.4% of patients, with a median of 16 days (IQR 11–20) from admission. ESBL-E RCS was performed in 86.2% of patients, with a positivity rate of 25.9%. The median time from admission to colonization detection was 6 days (IQR 0–14). The incidence of ESBL-E bacteremia was similar between carriers and non-carriers (5.6% vs. 2.5%, p=0.197). Comparisons between RCS and N-RCS were as follows: 1. CB use: 29.6% vs. 14%, p=0.003; 2. Appropriate EAT for *Enterobacterales* bacteremia: 81% vs. 83.3%, p=0.88; 3. In-hospital mortality: 6.2% vs. 4.5%, p=0.66. The number required to screen to predict ESBL-E bacteremia was 125.

**Conclusion:**

RCS for ESBL-E significantly increased CB use during the first episode of febrile neutropenia. However, it had no impact on the appropriateness of EAT or mortality. These findings suggest that routine ESBL-E colonization screening may not be required in HSCT patients.

**Disclosures:**

All Authors: No reported disclosures

